# Genome-wide transcriptomic analysis of the effects of sub-ambient atmospheric oxygen and elevated atmospheric carbon dioxide levels on gametophytes of the moss, *Physcomitrella patens*

**DOI:** 10.1093/jxb/erv197

**Published:** 2015-05-06

**Authors:** Suhas Shinde, Ali Behpouri, Jennifer C. McElwain, Carl K.-Y. Ng

**Affiliations:** ^1^School of Biology and Environmental Science, University College Dublin, Belfield, Dublin 4, Ireland; ^2^UCD Earth Institute, University College Dublin, Belfield, Dublin 4, Ireland

**Keywords:** Elevated carbon dioxide, microarray, *Physcomitrella patens*, sub-ambient oxygen.

## Abstract

It is widely accepted that atmospheric O_2_ has played a key role in the development of life on Earth, as evident from the coincidence between the rise of atmospheric O_2_ concentrations in the Precambrian and biological evolution. Additionally, it has also been suggested that low atmospheric O_2_ is one of the major drivers for at least two of the five mass-extinction events in the Phanerozoic. At the molecular level, our understanding of the responses of plants to sub-ambient O_2_ concentrations is largely confined to studies of the responses of underground organs, e.g. roots to hypoxic conditions. Oxygen deprivation often results in elevated CO_2_ levels, particularly under waterlogged conditions, due to slower gas diffusion in water compared to air. In this study, changes in the transcriptome of gametophytes of the moss *Physcomitrella patens* arising from exposure to sub-ambient O_2_ of 13% (oxygen deprivation) and elevated CO_2_ (1500 ppmV) were examined to further our understanding of the responses of lower plants to changes in atmospheric gaseous composition. Microarray analyses revealed that the expression of a large number of genes was affected under elevated CO_2_ (814 genes) and sub-ambient O_2_ conditions (576 genes). Intriguingly, the expression of comparatively fewer numbers of genes (411 genes) was affected under a combination of both sub-ambient O_2_ and elevated CO_2_ condition (low O_2_–high CO_2_). Overall, the results point towards the effects of atmospheric changes in CO_2_ and O_2_ on transcriptional reprogramming, photosynthetic regulation, carbon metabolism, and stress responses.

## Highlight

Transcriptomic analysis of the responses of *Physcomitrella patens* gametophytes to differential CO_2_ to O_2_ concentrations reveals extensive transcriptional reprogramming, photosynthetic acclimation, and altered oxidative signalling and defence responses.

## Introduction

The moss *Physcomitrella patens* is a non-vascular, multicellular land plant believed to have diverged from the land plant lineage more than 400 million years ago (Nishiyama *et al.*, 2003). *P. patens* has a relatively simple morphology and single-celled layer anatomy, thereby requiring constant co-equilibration of tissue water content with the environment (Reski, 1999; Quatrano *et al.*, 2007; Charron and Quatrano, 2009; Cho *et al.*, 2009). Such simple anatomical features imply the evolution of considerable intrinsic cellular and molecular mechanisms in response to abiotic stresses, and there is evidence to suggest that *P. patens* is highly tolerant of abiotic stresses (Frank *et al.*, 2005; Cho *et al.*, 2009; Mishler and Oliver, 2009; Koster *et al.*, 2010).

It is widely accepted that atmospheric O_2_ has played a key role in the development of life on Earth, as evident from the coincidence between the rise of atmospheric O_2_ concentrations in the Precambrian and biological evolution (Lenton, 2003, Taylor and McElwain, 2010). Our understanding of the variations in atmospheric O_2_ concentrations throughout the Phanerozoic is largely derived from models based on geochemical cycling of carbon and sulphur, with predictions of atmospheric O_2_ as low as 13–20% at times in the Mesozoic (Belcher and McElwain, 2008; Berner, 2009). It has been suggested that low atmospheric O_2_ is one of the major drivers for at least two of the five mass-extinction events in the Phanerozoic (Lenton, 2003; Huey and Ward, 2005).

Variations exist in the responses of plants to oxygen deprivation. For example, marine angiosperms such as *Zostera marina* (eelgrass) exhibit a high tolerance to oxygen deprivation (Pulido and Borum, 2010), and some species that inhabit marshes and wetlands can tolerate low oxygen availability for short periods (Luo *et al.*, 2012). At the molecular level, our understanding of the responses of plants to sub-ambient O_2_ concentrations is largely confined to studies of the responses of underground organs, e.g. roots, to hypoxic conditions (Bailey-Serres *et al.*, 2012), although developing embryos in seeds (particularly in large fruits) can also experience periods of oxygen deprivation (Crawford, 2012). Oxygen deprivation often results in elevated CO_2_ levels, particularly under waterlogged conditions, due to slower gas diffusion in water compared to air (Greenway *et al.*, 2006). When subjected to high concentrations of CO_2_, germinating chickpeas (*Cicer arietinum*) are less tolerant of oxygen deprivation (Crawford, 2012), suggesting an interaction between low O_2_ levels and elevated CO_2_ concentrations.

In this study, changes in the transcriptome of the *P. patens* arising from exposure to sub-ambient O_2_ (oxygen deprivation) and elevated CO_2_ were examined to further our understanding of the responses of non-vascular plants to changes in atmospheric composition. The results revealed previously unknown regulatory events associated with major physiological and biochemical responses to elevated CO_2_ or sub-ambient O_2_ alone, or in combination.

## Material and methods

### Plant growth

*P. patens* ecotype ‘Gransden 2004’ were propagated on cellophane overlay BCDAT agar plates under controlled conditions: photosynthetic photon flux density of 50 μmol s^-1^ m^-2^, 16h light/8h darkness, relative humidity of 75%, and 23°C in a Versatile Environmental Test Chamber, MLR-351 H (Sanyo, Japan). A mortar and pestle was used to homogenize 10–12-day-old moss protonemata in sterile distilled water. About 1–2mL of homogenized protonemal tissue was inoculated on sterile muslin cloth placed on top of sterile water-soaked Jiffy-7 peat pellets (Jiffy Products International AS, Kristansand, Norway) in GA7 Magenta boxes (Magenta Corporation, Chicago, IL, USA) containing 50–60mL sterile water. Adhesive microfiltration discs (18.6mm Ø, with inner filter area diameter of 10mm, and 2 μm pore size) (TQPL, Hampshire, UK) were pasted over a 5mm hole drilled through the lids used to close the G7 Magenta boxes. The containers were then sealed with Leukopor tape and incubated for 4–6 weeks under controlled conditions.

The 4–6-week-old *P. patens* gametophytes were transferred to Conviron BDW40 walk-in plant growth rooms (Conviron Europe Ltd., Isleham, UK) in University College Dublin Péac (Program for Experimental Atmospheres and Climate) and grown under the various conditions listed in Table 1. *P. patens* were acclimatized for 1 week under ambient CO_2_ and O_2_ conditions before being transferred to conditions of 1500 ppmV CO_2_ and ambient O_2_ (21%) (subsequently referred to as the ‘elevated CO_2_ treatment’), ambient CO_2_ (400 ppmV) and 13% O_2_ (subsequently referred to as the ‘sub-ambient O_2_ treatment’), and a combination of 1500 ppmV CO_2_ and 13% O_2_ (subsequently referred to as the ‘low O_2_–high CO_2_ treatment’), at a photosynthetic photon flux density of 50–70 μmol s^-1^ m^-2^, and 16h light/8h darkness. For control, gametophytes were kept on Jiffy-7 peat pellets in G7 Magenta boxes with microfiltration discs under ambient CO_2_ and O_2_ condition. Relative humidity within the chambers was maintained at 80% and chambers were set for midday peak temperature of 28°C and a night-time temperature of 18°C. *P. patens* gametophytes were grown under ambient and modified atmospheres for 7 days after which they were harvested at the same time and snap frozen in liquid N_2_ and stored at −80°C before RNA isolation. Special care was taken to avoid peat contamination.

**Table 1.** Summary of experimental conditions used in this study

Experimental condition	CO_2_ levels (ppm)	O_2_ levels (ppm)	CO_2_ to O_2_ ratio	CO_2_ to O_2_ ratio relative to ambient CO_2_ and O_2_ levels
Ambient	400	210 000	0.0019:1	1:1
Elevated CO_2_	1500	210 000	0.0071:1	3.75:1
Sub-ambient O_2_	400	130 000	0.0030:1	1:0.619
Low O_2_–high CO_2_	1500	130 000	0.0115:1	3.75:0.619

### CO_2_ measurement using gas chromatography

Gas chromatography analyses were conducted for gas samples from within the GA7 Magenta containers where the *P. patens* were grown to determine the efficiency of gas exchange via the adhesive microfiltration discs fixed on the container lids. CO_2_ analysis was carried out on a gas chromatograph (Shimadzu GC-2014; Shimadzu Europa GmbH, Duisburg, Germany) fitted with an electron capture detector (carrier gas N_2_ at a flow rate of 20mL min^-1^) with an automated injection system (Loftfield *et al.*, 1997). The gas peak area was recorded with the Peaksimple software (SRI Inc., Menlo Park, CA, USA) and used for determination of CO_2_ concentrations. The concentration of CO_2_ within the Magenta GA7 containers corresponded to the CO_2_ concentration within the growth chamber, indicating efficient gas exchange of gases via the adhesive microfiltration disc. CO_2_ concentration is used here as a proxy for efficiency of gas exchange via the adhesive microfiltration discs.

### Total RNA isolation

Total RNA was extracted from *P. patens* gametophytes using the RNeasy Mini Kit (Qiagen, Manchester, UK) according to the protocol recommended by the manufacturer. Total RNA was eluted with 30 to 50 μL of RNAse-free water. Qualities and quantities of total RNA were determined using a Nanodrop ND 1000 Spectrophotometer (Nanodrop Technologies, Hemel Hempstead, UK). Total RNA integrity was determined with an Agilent 2100 BioAnalyzer (Agilent Technologies, Inc., Santa Clara, CA, USA).

### Microarray analysis

Microarray analyses were conducted using a two-colour loop design (Churchill, 2002) to compensate for dye bias as the amount of label per amount of RNA is different for Cy3 and Cy5, by making duplicate hybridizations with the same sample using both Cy3 and Cy5 labelling and averaging the ratios from dye-swapped hybridizations. Three biological replicates from each experimental condition were analysed in duplicate by MOgene LC (St. Louis, MO, USA), a Agilent-certified service provider specializing in *P. patens* microarray analysis. The chip used was a 60-base oligomer cDNA array comprising a total of 22 895 ESTs generated by Leeds University, UK, and the National Institute for Basic Biology, Japan (Cuming *et al.*, 2007; Rensing *et al.*, 2008).

Total RNA (2.5 μg) was labelled using the ULS-Cy 3/5 ULS RNA Labelling Kit (Product #EA-006; Kreatech Biotechnology, San Diego, CA, USA). Following labelling of RNA with Cy3 and Cy5 fluorescent dyes, equal amounts of RNA (1 μg) were mixed in nuclease-free water and processed using the Gene Expression Hybridization Kit (Product #5188–5242; Agilent Technologies Inc.). For hybridization, samples were placed between the Agilent backing slide and microarray chip, sealed in the hybridization chamber, and hybridized for 17h in a 60°C rotating hybridization oven. After hybridization, the slides were washed sequentially in 6× SSC buffer (0.15M NaCl, 15nM Na-acetate, pH 7.0) at room temperature, then 0.1× SSC on ice. Nitrogen gas was used to dry the slides before scanning using a DNA Microarray Scanner (#G2565BA; Agilent Technologies Inc.) with the Agilent Scan Control software. The fluorescent intensities of each feature were extracted using Feature Extraction Software with default parameters (Ver. 9.1, Agilent Technologies Inc.). The raw intensity data were then log (base e = 2.718) transformed for normalization before ANOVA (mixed model) analysis. When the raw intensities of both Cy3 and Cy5 channels were below 150 (typical background intensity is 40) and the signal-to-background ratio below 2, genes were removed from further analysis. Log ratios among different samples and the *P*-values were calculated using a mixed model (Wolfinger *et al.*, 2001). A significant change in gene expression was based on the 2-fold cut-off with *P*-values <0.05 (Cuming *et al.*, 2007).

Microarray data were normalized against ambient condition (ambient CO_2_ and O_2_) to examine the effect of different concentrations of CO_2_ and O_2_ on gene expression in *P. patens* gametophytes. Recent annotations of the *Physcomitrella* gene models were retrieved from the Department of Energy Joint Genome Institute (http://genome.jgi-psf.org/Phypa1_1/Phypa1_1.home.html) (Zimmer *et al.*, 2013). Functional annotations based on BLAST2GO analysis were used for a detailed analysis of the response of *P. patens* gametophytes to the different experimental atmospheres in this study.

### Semi-quantitative reverse transcriptase PCR

Reverse transcriptase (RT)-PCR was conducted as previously described (Xiong *et al.*, 2009; O’Donoghue *et al.*, 2013). Briefly, total RNA was isolated using the Qiagen RNeasy kit according to the manufacturer’s recommendations. The quality and quantity of the total RNA was determined using a NanoDrop 1000 spectrophotometer. One microgram of total RNA was treated with 1U of DNase I (Invitrogen, Hemel Hempstead, UK) before being used for cDNA synthesis using 200U of M-MLV Reverse Transcriptase (Invitrogen). The resultant cDNA was used for PCR amplification using 0.5U of Go-Taq DNA polymerase (Promega, Southampton, UK). The sequences of the primers used for amplification are listed in Supplementary Table S1.

## Results and discussion

In this study, the effects of a 7-day exposure to elevated CO_2_ (1500 ppmV), sub-ambient O_2_ (13%), and low O_2_–high CO_2_ [combination of elevated CO_2_ (1500 ppmV) and sub-ambient O_2_ (13%)] on transcriptomic changes in *P. patens* gametophytes were examined. No observable morphological changes were observed in *P. patens* gametophytes under these modified atmospheric conditions (data not shown).

### Elevated CO_2_ evoked large-scale transcriptome response in *P. patens* gametophytes

**Fig. 1.**
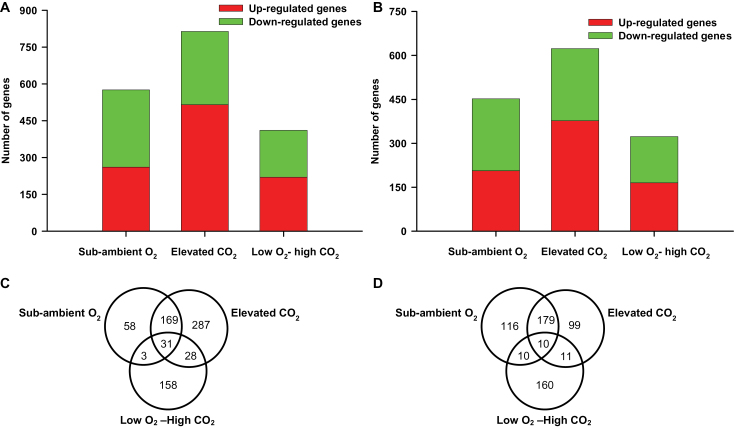
Microarray analysis of the number of genes whose expression are affected in *P. patens* gametophytes following a 7-day exposure to sub-ambient O_2_ (13%), elevated CO_2_ (1500 ppmV), and low O_2_–high CO_2_ compared to ambient CO_2_ and O_2_ level. **(A)** The number of transcripts exhibiting significant changes, relative to control ambient conditions (fold change ≥ 2; *P* ≤ 0.05, red and green indicates number of up- or down-regulated genes respectively) are indicated. **(B)** Numbers of *P. patens* transcripts homologous to *Arabidopsis* genes are indicated. **(C)** Venn diagram showing the number of *P. patens* genes commonly or exclusively up-regulated and **(D)** down-regulated in response to elevated CO_2_ (1500 ppmV), sub-ambient O_2_ (13%), and low O_2_–high CO_2_ compared to control ambient environment.

Microarray analysis revealed that the expression of a large number of genes (n = 814, fold change ≥ 2; *P* < 0.05), relative to ambient conditions (control), were significantly affected when *P. patens* gametophytes were grown under elevated CO_2_ conditions. The expression of a relatively smaller number of genes was observed under sub-ambient O_2_ and low O_2_–high CO_2_ treatment, in which a total of 576 and 411 genes were significantly affected, respectively (Fig. 1A). Of the 814 genes significantly affected under elevated CO_2_, 63% were up-regulated, whereas only 45% and 54% of the transcripts were up-regulated in response to sub-ambient O_2_, and low O_2_–high CO_2_ treatment, respectively (Fig. 1A). Interestingly, the capacity of elevated CO_2_ to up-regulate gene expression in *P. patens* gametophytes was reduced by half when atmospheric O_2_ content was reduced from 21% to 13% (Fig. 1A), suggesting a possible interaction between atmospheric concentrations of CO_2_ and O_2_ on plant responses. Of the 814 genes expressed in response to elevated CO_2_, a total of 623 (377 up-regulated and 246 down-regulated) genes showed homology to annotated genes from *Arabidopsis thaliana* (Fig. 1B). Out of 576 expressed genes in response to sub-ambient O_2_, 206 up-regulated and 246 down-regulated genes showed homology with *A. thaliana* genes. In low O_2_–high CO_2_ treatment, out of 411 expressed genes, 165 up-regulated and 158 down-regulated *Physcomitrella* genes showed homology with *A. thaliana* genes (Fig. 1B).

There was considerable overlap between the sets of genes up-regulated in sub-ambient O_2_ and elevated CO_2_ of 169 genes. Only 3 up-regulated genes showed overlap between sub-ambient O_2_ and low O_2_–high CO_2_ treatment. Elevated CO_2_ and low O_2_–high CO_2_ treatment shared a set of 28 up-regulated genes (Fig. 1C). Of the genes analysed, 58 genes were exclusively up-regulated under sub-ambient O_2_, whereas the expression of larger numbers of genes were specifically up-regulated under elevated CO_2_ (287 genes), and low O_2_–high CO_2_ treatment (158 genes) (Fig. 1C). Thirty-one genes were up-regulated in all three conditions. There was also a significant overlap between the set of genes down-regulated in the various atmospheres (Fig. 1D). The response to elevated CO_2_ and sub-ambient O_2_ was strikingly similar in moss gametophytes, with 179 genes commonly down-regulated. Expression was down-regulated in a greater number of genes under low O_2_–high CO_2_ treatment (160 genes) than under elevated CO_2_ (99 genes) and sub-ambient O_2_ (116 genes) (Fig. 1D).

**Fig. 2.**
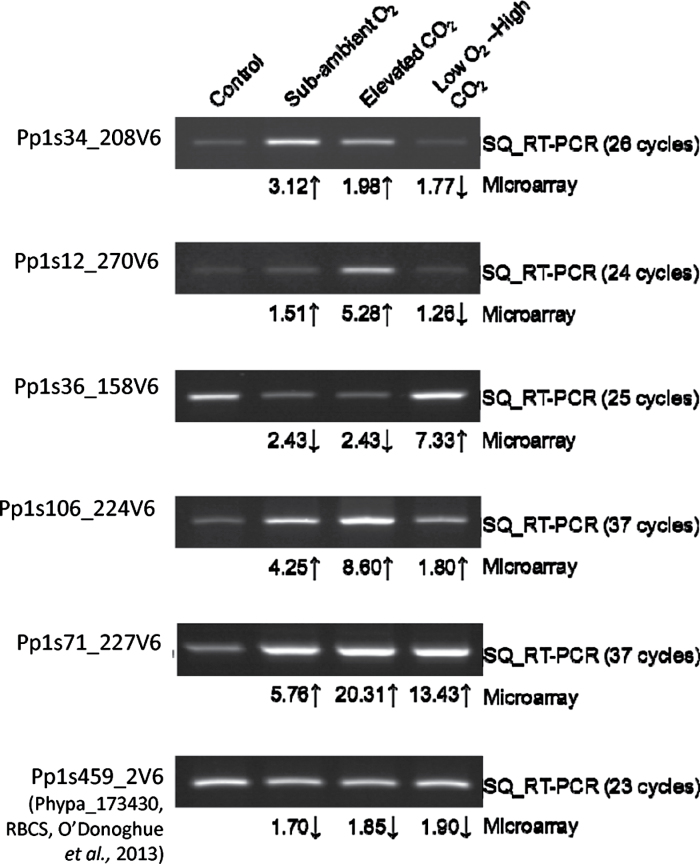
RT-PCR validation of microarray data. RNA was extracted from gametophytes and semi-quantitative RT-PCR was performed using primers designed to amplify a selection of genes identified as being differentially expressed in microarray experiments under conditions of sub-ambient O_2_, elevated CO_2_, and low O_2_–high CO_2_. The corresponding fold-change as identified from microarray experiments is indicated below each gel band. Amplified DNA was run on a 1% (w/v) agarose gel and stained with ethidium bromide before visualization under UV illumination. Images shown are representative of three independent biological replicates.

To validate the microarray data, a selection of genes identified as being differentially expressed in microarray experiments under conditions of sub-ambient O_2_, elevated CO_2_, and low O_2_–high CO_2_ treatment were chosen for semi-quantitative RT-PCR analysis. The results of semi-quantitative RT-PCR showed expression patterns consistent with data from the microarray dataset (Fig. 2). In agreement with O’Donoghue *et al.* (2013), who successfully used semi-quantitative RT-PCR to validate their microarray dataset from *P. patens*, the semi-quantitative RT-PCR data presented here clearly show that the microarray data can be used to indicate changes in gene expression levels in *P. patens* gametophytes under the various atmospheric conditions examined (Fig. 2).

MapMan molecular functional classification analysis was performed to understand the overall transcriptomic response in *P. patens* to the different atmospheres using the corresponding *A. thaliana* locus IDs (AGIs) as a reference to categorize differentially expressed *P. patens* genes using a web-based interface, The Bio-Array Resource (http://bar.utoronto.ca/welcome.htm) (Provart and Zhu, 2003). Approximately half of the *P. patens* genes possess homologues in *A. thaliana* (data not shown). Significantly expressed genes were classified into 31 different functional categories. The data suggest that the expression of genes involved in metabolic processes, stress, photosynthesis and transport functions are significantly altered in response to all 3 atmospheric conditions (Supplementary Fig. S1).

### Genes implicated in transcriptional regulation

Microarray results showed that the expression of large numbers of transcription factors was significantly regulated in response to elevated CO_2_. A total of 16 and 12 transcripts encoding transcription factors were up- and down-regulated, respectively (Supplementary Table S2). MYB transcription factor family proteins, zinc-binding family proteins, and Integrase-type DNA-binding superfamily protein were among the highly expressed transcription-related genes. Interestingly, there was a significant overlap between up- and down-regulated transcription factor genes in response to sub-ambient O_2_ and elevated CO_2_. Of the 12 up-regulated and 8 down-regulated genes under sub-ambient O_2_ (13%), a set of 10 up-regulated and 3 down-regulated genes encoding transcription factors showed common expression patterns with genes expressed under elevated CO_2_ (Supplementary Table S2 and Supplementary Fig. S2). The effects of a low O_2_–high CO_2_ condition were small, with only one and five genes encoding transcription factors in *P. patens* exclusively up- and down-regulated, respectively (Supplementary Table S2). Together, these data suggest transcriptional reprogramming in response to a 7-day exposure to modified atmospheres, with elevated CO_2_ and sub-ambient O_2_ conditions eliciting a greater extent of transcriptional reprogramming compared to a 7-day exposure to a low O_2_–high CO_2_ treatment (Supplementary Table S2 and Supplementary Fig. S2). It is unclear why exposure to a low O_2_–high CO_2_ treatment resulted in a lesser extent of transcriptional reprogramming. However, it is possible that plants have not, throughout their evolutionary history, experienced this unique atmospheric condition (low O_2_–high CO_2_) and, as such, have not evolved the necessary regulatory transcriptional mechanism(s) to respond effectively.

### Differential expression of photosynthetic and carbon metabolism genes

**Fig. 3.**
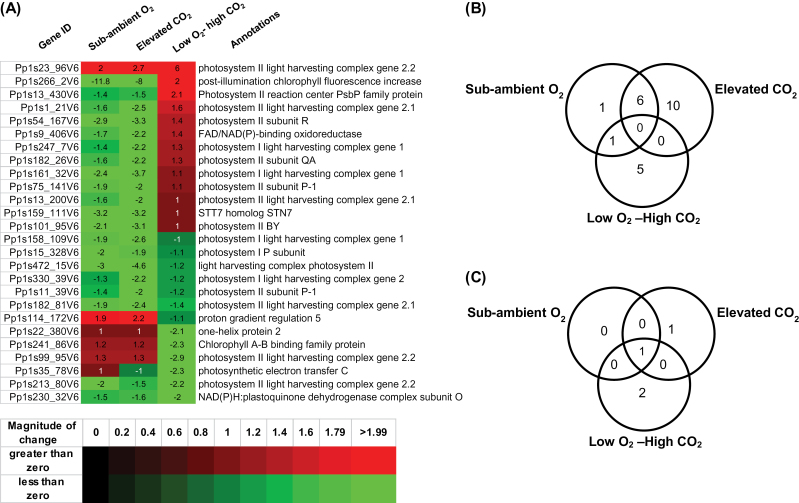
Differentially expressed *P. patens* genes involved in photochemical reactions of photosynthesis in response to elevated CO_2_ (1500 ppmV), sub-ambient O_2_ (13%), and low O_2_–high CO_2_. (A) Heat map of the significantly expressed transcripts of photosynthesis based on MapMan functional classification. (B) Venn diagram showing overlapping transcripts that are significantly down-regulated in *P. patens* gametophytes exposed to elevated CO_2_, sub-ambient O_2_, or low O_2_–high CO_2_ treatment. (C) Venn diagram showing overlapping transcripts that are significantly up-regulated in *P. patens* gametophytes exposed to elevated CO_2_, sub-ambient O_2_, or low O_2_–high CO_2_ treatment.

Differential expression of genes encoding proteins involved in photosynthesis and carbohydrate metabolism-related functions was also observed, suggesting the existence of a metabolic response strategy to elevated CO_2_ or sub-ambient O_2_ individually, or low O_2_–high CO_2_ treatments (Fig. 3) in *P. patens* gametophytes. Elevated atmospheric CO_2_ can exert profound effects on photosynthesis in many plants (Stitt and Krapp, 1999; Long *et al.*, 2004; Dermody *et al.*, 2008; Leakey *et al.*, 2009). Bryophytes generally obtain CO_2_ by diffusion and are not limited by opening and closing of stomata. Atmospheric CO_2_ diffuses through the cell wall into the cytosol and dissolves in cell wall or apoplast water to form bicarbonate (HCO_3_^-^). It is made available to the site of CO_2_ consumption by the primary carboxylating enzyme, ribulose-1,5-carboxylase/oxygenase (RuBP) in the chloroplast stroma (Price *et al.*, 2011). Under elevated CO_2_, an initial increase in the rate of carbon fixation was observed in many C3 plants, resulting in the accumulation of soluble sugars such as glucose, fructose, and sucrose, which are the main products of photosynthetic carbon assimilation (Moore *et al.*, 1997; Ainsworth, 2008). Reduced photosynthetic capacity under long-term exposure to high CO_2_ is mainly attributed to increased accumulation of storage carbohydrates (Chen *et al.*, 2005; Ainsworth and Long, 2005). This down-regulation or inhibition of photosynthetic activity is generally referred to as the acclimation responses of plants to elevated levels of atmospheric CO_2_ (Stitt and Krapp, 1999). There is evidence indicating that increased levels of CO_2_ can modulate expression of several photosynthesis- and Calvin-cycle–related genes (Rogers *et al.*, 1996; Gesch *et al.*, 1998; Moore *et al.*, 1999; Fukayama *et al.*, 2009). Here, *P. patens* orthologues were identified for many annotated *Arabidopsis* transcripts functionally associated with the photochemical reactions of photosynthesis, e.g. the light-harvesting complex LHCII. In photosynthetic organisms, LHCII absorbs and transfers excitation energy to the photosystem antenna (Galka *et al.*, 2012). A total of 16 LHCII-related genes were significantly down-regulated under elevated CO_2_ whereas only eight LHCII genes were down-regulated under sub-ambient O_2_, of which six were commonly repressed in both conditions. Expression of three transcripts was repressed in response to low O_2_–high CO_2_ treatment (Fig. 3A,B; Supplementary Table S3). Only three light reaction-related genes were up-regulated in *P. patens* gametophytes exposed to a low O_2_–high CO_2_ treatment. One gene, Pp1s23_96V6, encoding a chlorophyll a-b binding protein of LHCII type I protein showed induced expression in all three experimental conditions. Moreover, expression of only one and two light reaction-related genes were induced independently in elevated CO_2_ and low O_2_-–high CO_2_ treatment, respectively (Supplementary Table S3). The data suggest a reduction in photochemical reactions of photosynthesis in *P. patens* gametophytes under elevated CO_2_ conditions. However, the expression of genes encoding proteins involved in light reactions was not significantly affected in *P. patens* gametophytes under low O_2_–high CO_2_ treatment, indicating that photochemical reactions of photosynthesis are likely to be functional in this environmental regime in moss gametophytes.

The effect of different atmospheric conditions on expression of genes involved in CO_2_ fixation, RuBP regeneration, and starch synthesis in moss gametophytes was also examined. Under elevated CO_2_ treatment, the expression of a total of 21 genes involved in CO_2_ fixation, RuBP regeneration, and starch biosynthesis was altered. Expression of a set of 14 and 26 genes involved in CO_2_ fixation, RuBP regeneration, and starch biosynthesis was altered in response to sub-ambient O_2_ and low O_2_–high CO_2_ treatments, respectively (Table 2, Supplementary Fig. S3). Interestingly, diverse transcriptome responses by moss gametophytes to the low O_2_–high CO_2_ treatment condition were observed. Differential expression of a greater number of transcripts (total 13: 4 up-regulated and 9 down-regulated) was specifically detected upon exposure to the low O_2_–high CO_2_ treatment condition (Supplementary Fig. S3). These down-regulated *P. patens* transcripts encode for major components of CO_2_ fixation: carbonic anhydrase, a protein that catalyses reversible conversion of CO_2_ to HCO_3_^-^; RuBP small subunit; glyceraldehyde-3-phosphate dehydrogenase; and RuBP activase (Table 2). Overall, 19 genes encoding proteins related to CO_2_ fixation and RuBP regeneration were down-regulated in *P. patens* gametophytes under low O_2_–high CO_2_ treatment (Table 2). Six transcripts encoding enzymes of starch synthesis, such as ADP-glucose pyrophosphorylase (AGPase) and starch synthase, were highly expressed in *P. patens* gametophytes under low O_2_–high CO_2_ treatment compared to sub-ambient O_2_ and elevated CO_2_ (Table 2), suggesting the accumulation of soluble sugars in *P. patens* gametophytes. For example, *P. patens* gene Pp1s98_52V6, encoding a glucose-1-phosphate adenylyltransferase large subunit (AGPase), showed greater than 19-fold down-regulation upon exposure to sub-ambient O_2_, and 13-fold down-regulation to elevated CO_2_, while expression of the same gene was strongly induced (≥ 4-fold) under low O_2_–high CO_2_ treatment (Table 2). Exposure to the low O_2_–high CO_2_ condition resulted in elevated expression of genes associated with starch metabolism and starch degradation in moss gametophytes; whereas starch metabolism was partially affected under sub-ambient O_2_ and elevated CO_2_ conditions (Table 2). In photosynthetic organisms, photosynthesis is controlled by a sugar-dependent metabolic feedback mechanism. Carbohydrate-dependent feedback inhibition on photosynthetic activity represses expression of RuBP small subunit, RuBP activase, and chlorophyll a/b binding proteins (Gesch *et al.*, 1998; Moore *et al.*, 1999; Stitt and Krapp 1999; Chen *et al.*, 2005). The results suggest that expression of genes functioning in photosynthetic carbon fixation and RuBP regeneration activity may be affected by feedback inhibition mechanisms due to accumulation of soluble sugars. In plants, sugars are important components of the sugar sensing and signalling network that regulate plant growth and development (Rolland *et al.*, 2006). Sugar availability modulates global gene expression patterns via highly complex mechanisms controlling transcription, translation, and protein stability (Rolland *et al.*, 2006). Transcriptome profiling indicates differential responses of *P. patens* gametophytes at the molecular level to elevated CO_2_, sub-ambient O_2_, and low O_2_–high CO_2_ treatments. The Hexokinase 1 (HXK1) protein is identified as one of the conserved sugar sensors implicated in controlling expression of genes involved in primary carbon fixation and RuBP regeneration in plants (Jang *et al.*, 1997, Xiao *et al.*, 2000, Rolland *et al.*, 2006).

**Table 2.** *Altered expression of* P. patens *genes involved in CO_2_ fixation, RuBP regeneration and starch synthesis in response to sub-ambient O_2_, elevated CO_2_ and low O_2_-high CO_2_*

Function	Enzymes	Gene ID	Sub-ambient O_2_	Elevated CO_2_	Low O_2_-high CO_2_
CO_2_ fixation	Carbonic anhydrase	Pp1s264_55V6	-2.64	-2.45	-2.07
		Pp1s43_118V6	-	-	-2.46
		Pp1s30_234V6	-	-	2.34
	RuBP small subunit	Pp1s204_93V6	-2.09	-2.07	-2.24
		Pp1s188_39V6	2.6	2.54	-2.61
		Pp1s374_50V6	-	-	-2.4
		Pp1s66_48V6	-	-	-2.19
		Pp1s545_4V6	-	-	-2.7
	RuBP activase	Pp1s5_83V6	-	-2.02	-2.06
		Pp1s258_44V6	-	-2.11	-2.14
		Pp1s199_130V6	-	-	-3.09
		Pp1s199_129V6	-	-	-2.69
	GAPDH	Pp1s10_228V6	2.42	2.01	2.52
		Pp1s135_21V6	-2.81	-2.42	-
		Pp1s9_47V6	-2.29	-	-
		Pp1s49_34V6	-	2.98	-
		Pp1s414_8V6	-	-2.22	-3.36
RuBP regeneration	SBPase	Pp1s429_29V6	-	-2.15	-2.07
	FBPase	Pp1s385_43V6	-2.07	-	-
		Pp1s242_66V6	-	-2.12	-2.24
		Pp1s163_36V6	-	-	3.18
	Aldolase	Pp1s50_50V6	-	-	-2.86
		Pp1s475_27V6	-	-	-2.93
		Pp1s33_389V6	-	-	-2.1
Starch synthesis	Starch synthase	Pp1s124_155V6	-4.76	-4.39	13.98
		Pp1s234_74V6	-2.02	-2.61	-
		Pp1s93_98V6	-	2.86	-
		Pp1s150_86V6	-	-2.46	-
		Pp1s302_51V6	-	-	3.3
		Pp1s104_136V6	-	-	2.26
	GBSS	Pp1s12_341V6	-	-2.52	-
	ADP-GPP	Pp1s397_21V6	4.07	2.07	11.61
		Pp1s389_5V6	-2.57	-2.53	-
		Pp1s98_52V6	-19.99	-13.98	4.59
		Pp1s36_158V6	-2.43	-2.43	7.33
		Pp1s2_204V6	3.28	2.73	-

ADP-GPP, ADP-glucose pyrophosphorylase; GAPDH, glyceraldehyde-3-phosphate dehydrogenase; GBSS, granule-bound starch synthase.

Differentially altered expression of two *P. patens* transcripts encoding for HXK1 protein was observed. Transcript abundance of *P. patens* gene Pp1s12_19V6 encoding HXK1 protein was significantly increased in response to low O_2_–high CO_2_ treatments (Supplementary Table S4A). Another transcript, Pp1s414_10V6, encoding HXK1 protein was highly repressed under sub-ambient O_2_ and elevated CO_2_ conditions. In higher plants, sugar-induced elevation of calcium-dependent protein kinases, SNF1-related kinases, and Ca^2+^-fluxes are associated with sugar signalling where Ca^2+^ acts as a second messenger (Rolland *et al.*, 2006). The data presented here revealed down-regulation (≥ 6-fold) of SNF1 kinase homolog 11 gene (Pp1s107_182V6) in response to both sub-ambient O_2_ and elevated CO_2_ treatments (Supplementary Table S4A). One calcium-dependent protein kinase gene (Pp1s309_91V6) was induced under elevated CO_2_. Accumulation of two CBL-interacting serine/threonine-protein kinase genes (Pp1s58_13V6 and Pp1s79_209V6) was detected in gametophytes subjected to sub-ambient O_2_ and elevated CO_2_ treatments (Supplementary Table S4A).

Elevations in transcript abundance of the five genes encoding enzymes of glycolysis were observed only in the low O_2_–high CO_2_ condition (Supplementary Table S4B). Significant overlap between the sets of genes expressed (one up-regulated and two down-regulated) in sub-ambient O_2_ and elevated CO_2_ was observed, suggesting that moss gametophytes responded similarly to the sub-ambient O_2_ or elevated CO_2_ treatments at the molecular level. This was supported by another identical expression pattern of genes involved in glycolysis (Supplementary Table S4B). More elevated transcript abundance of the genes encoding enzymes of glycolysis was observed only in the low O_2_–high CO_2_ condition (Supplementary Table S4B), indicating that long-term exposure to a higher CO_2_ to O_2_ ratio may have triggered foliar respiration in moss gametophytes due to higher substrate availability (Davey *et al.*, 2004; Ainsworth *et al.*, 2006).

### Membrane transporters

Large-scale changes in the *P. patens* transcriptome occurred in response to elevated CO_2_. A large number of transcripts (n = 31) involved in membrane transport functions was significantly up-regulated in response to the elevated CO_2_ condition compared to the sub-ambient O_2_ and low O_2_–high CO_2_ conditions, where 12 and 8 genes were up-regulated respectively (Supplementary Table S5A). These membrane transport–related *P. patens* genes were homologues of *Arabidopsis* transporter genes such as NRAMP metal ion transporter 4, ABC transporter family protein, glutathione S-conjugate transporter, and calcium-transporting ATPase. A total of 17, 15, and 13 genes were down-regulated under the sub-ambient O_2_, elevated CO_2_, and low O_2_–high CO_2_ treatments, respectively (Supplementary Table S5B). Of the 31 up-regulated genes in response to elevated CO_2_ treatment, 22 genes were uniquely expressed in this condition (Supplementary Fig. S4 and Supplementary Table S5A). Interestingly, all eight genes up-regulated in the low O_2_–high CO_2_ treatment were exclusively expressed (Supplementary Fig. S4). In the sub-ambient O_2_ treatment, of the 12 up-regulated membrane transporter genes, nine genes showed overlap with genes up-regulated in the elevated CO_2_ condition (Supplementary Fig. S4 and Supplementary Table S5A). These data suggest that elevated CO_2_ intensified expression of membrane transport function. By contrast, exposure to the low O_2_–high CO_2_ condition had significantly less effect on the expression of transporter genes in gametophytes.

### Effects on hormone metabolism and signal transduction

**Fig. 4.**
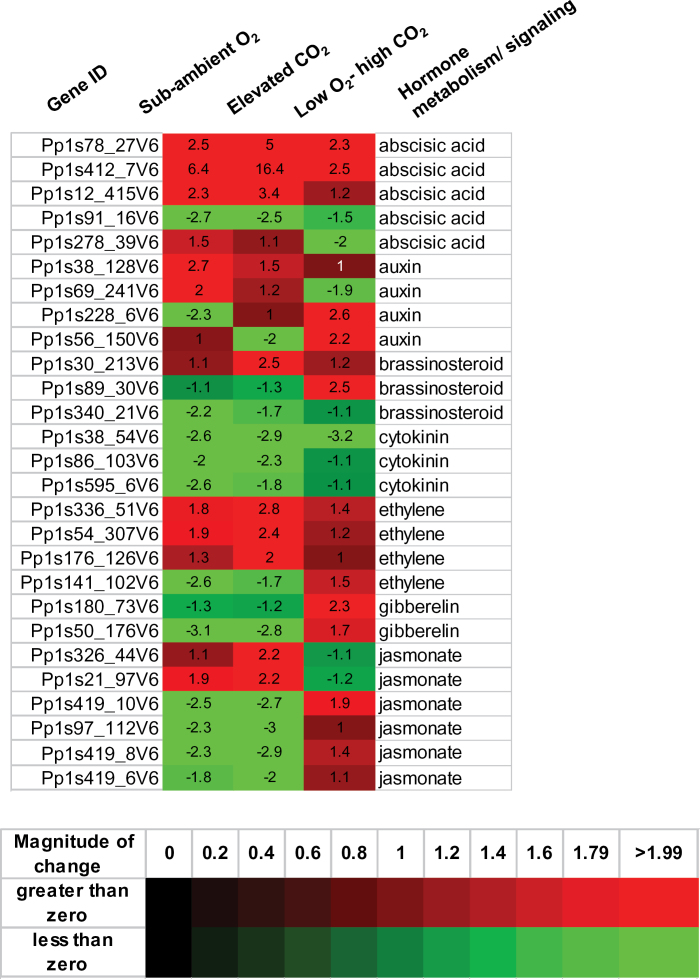
Effects of elevated CO_2_, sub-ambient O_2_ and low O_2_-high CO_2_ treatments on expression of genes involved in hormone metabolism in *P. patens* gametophytes. Heat map of the significantly up and down-regulated genes based on MapMan functional classification.

The impact of changing atmospheric CO_2_ levels on plant hormone metabolism is not well understood (Ribeiro *et al.*, 2012). Plant hormones play vital roles in metabolic adjustment and modulation of gene expression under various environmental conditions to maintain plant growth and development (Bohnert *et al.*, 1995; Kempa *et al.*, 2008). Abscisic acid (ABA) plays a central role in adaptive responses to various abiotic stresses in plants. The ABA biosynthetic pathway in plants is regulated by 9-cis-epoxycarotenoid dioxygenase (NCED) genes. Markedly induced transcript levels of *NCED9* (Pp1s412_7V6) were observed in microarray analysis in response to elevated CO_2_ (fold change ≥ 16) and sub-ambient O_2_ (fold change ≥ 6) compared to the low O_2_–high CO_2_ condition (fold change ≥ 2) (Supplementary Table S6), suggesting that the ABA pool may have increased in moss gametophytes grown in elevated CO_2_ and sub-ambient O_2_ conditions. Notably, expression of key genes involved in metabolism of hormones such as auxin, brassinosteroid, cytokinin, ethylene, gibberellin, and jasmonate was altered significantly in moss gametophytes exposed to these different atmospheric conditions (Supplementary Table S6). Heat-map analysis of expressed *P. patens* genes of hormone metabolism demonstrated differential molecular responses to elevated CO_2_ and sub-ambient O_2_ individually and in combination (Fig. 4). MapMan ontology analysis revealed that exposure to elevated CO_2_ intensified up-regulation of ABA, brassinosteroid, and ethylene metabolism-related genes (Fig. 4 and Supplementary Table S6). In plants, jasmonic acid, a lipid-based hormone that regulates anti-herbivore defences, is a product of the octadecanoid pathway (Wasternack, 2007). Expression of two genes encoding for 12-oxophytodienoate reductase activity (*OPR1* and *OPR2*) was strongly up-regulated and 4 lipoxygenase 3 (*LOX3*) genes the in octadecanoid pathway were down-regulated, indicating that jasmonate metabolism was also altered by elevated CO_2_ treatment (Supplementary Table S6). However, elevated CO_2_ significantly dampened expression of genes involved in gibberellin, cytokinin, and auxin metabolism (Fig. 4 and Supplementary Table S6). Upon exposure to sub-ambient O_2_ conditions, methylesterase (associated with auxin biosynthesis) and an O-fucosyltransferase-like protein gene involved in cytokinin metabolism were highly expressed in moss gametophytes (Supplementary Table S6), whereas expression of ethylene-, brassinosteroid-, cytokinin-, gibberellin-, and jasmonate-related genes was down-regulated. Interestingly, ABA-, auxin-, and gibberellin-related genes were partially induced or repressed in moss gametophytes grown under the low O_2_–high CO_2_ condition (Supplementary Table S6). Additionally, in contrast to elevated CO_2_ and sub-ambient O_2_ treatments, exposure to low O_2_–high CO_2_ treatment had no effect on expression of ethylene metabolism– or ethylene signalling–related genes (Fig. 4 and Supplementary Table S6). These results suggest that elevated CO_2_ induced reprogramming of hormone metabolism in moss gametophytes and highlight the differential molecular acclimation potential of *P. patens* gametophytes to changing environmental conditions.

### Elevated CO_2_ triggers expression of stress-related genes

**Fig. 5.**
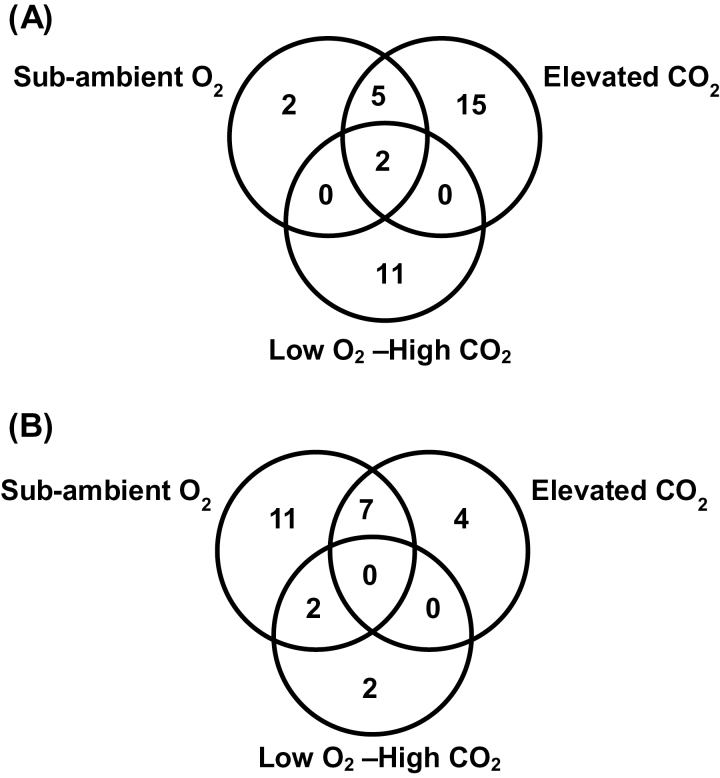
Effects of elevated CO_2_, sub-ambient O_2_, and low O_2_–high CO_2_ treatments on expression of stress-related genes in *P. patens* gametophytes. Venn diagram represents number of commonly and distinctly (A) up-regulated and (B) down-regulated genes.

MapMan analysis of *P. patens* genes using *Arabidopsis* homologues as a reference revealed significant alteration in stress-related transcripts (Supplementary Fig. S1). Thirty-three stress-related genes responded to elevated CO_2_ (22 up-regulated and 11 down-regulated) (Fig. 5A,B and Supplementary Table S7), while altered expression of 29 stress-related transcripts (9 up-regulated and 20 down-regulated) was observed in moss gametophytes exposed to sub-ambient O_2_ (Supplementary Table S7). By contrast, only 17 (13 up-regulated and 4 down-regulated) stress-associated genes were expressed in response to the low O_2_–high CO_2_ condition (Supplementary Table S7). The expression of a comparatively large number of stress- and defence-related genes was repressed in sub-ambient O_2_ and elevated CO_2_ conditions (Fig. 5B). We observed an increase in the transcript abundance of the ABA biosynthesis enzyme, PpNCED9 (≥ 16-fold) upon elevated CO_2_ treatment, suggesting that ABA levels are likely to have increased in moss gametophytes. ABA accumulation in plants under stress conditions induces transcriptional reprogramming, eliciting many responses at the physiological, biochemical, and molecular levels (Shinozaki and Yamaguchi-Shinozaki, 1996). Application of exogenous ABA induced rapid transcriptional responses in *P. patens* protonemata (Cuming *et al.*, 2007; Richardt *et al.*, 2010 Shinde *et al.*, 2012), and induces stress responses and stress tolerance in *P. patens* (Frank *et al.*, 2005; Khandelwal *et al.*, 2010; Shinde *et al.*, 2012). Proteins involved in stress signalling and stress tolerance were highly accumulated in *P. patens* gametophytes after ABA treatment (Wang *et al.*, 2009, Cui *et al.*, 2012). In this study, it was observed that transcripts involved in stress-related functions were highly induced upon elevated CO_2_ treatment (Supplementary Table S7). Evidence for a triggered transcriptional response of stress-response genes in moss gametophytes grown under elevated CO_2_ can potentially be attributed to the increased expression of transcripts encoding a key enzyme in the ABA biosynthetic pathway.

Recently, increased oxidative stress in plants grown under elevated CO_2_ was reported (Qiu *et al.*, 2008, Gillespie *et al.*, 2011). In C3 plants, despite expected reduced levels of reactive oxygen species under elevated CO_2_, oxidative signalling is emerging as an unexpected component of plant response to elevated CO_2_. Antioxidant enzymes act as scavengers and are associated with cellular detoxification of reactive oxygen species during oxidative stress (Mittler, 2002; 2006). Significant up-regulation of genes encoding enzymes associated with oxidative stress response and signalling and redox regulation was observed in *P. patens* gametophytes subjected to elevated CO_2_ treatment (Table 3). A total of 13 and 11 genes encoding for these enzymes and genes with putative functions in detoxification were up-regulated under elevated CO_2_ and low O_2–_high CO_2_ treatment respectively. These genes included peroxidase, catalase, 12-oxophytodienoate reductase, and glutathione s-transferase (Table 3). Only three genes, Pp1s34_208V6 (L-galactono-1,4-lactone dehydrogenase), Pp1s98_250V6 (GDP-D-mannose 3′,5′-epimerase), and Pp1s396_10V6 (SOUL heme-binding family protein), involved in redox balance were up-regulated in *P. patens* under sub-ambient O_2_ treatment (Table 3). Additionally, a set of 11 genes mainly encoding peroxidase superfamily protein, thioredoxin superfamily protein, and catalase 1 were significantly up-regulated in *P. patens* gametophytes under low O_2_–high CO_2_ condition. Antioxidant enzymes such as catalase, dehydroascorbate reductase, glutathione-dependent formaldehyde dehydrogenase, and peroxidase were among the highly expressed *P. patens* genes under this condition (Table 3). Interestingly, only a single transcript coding for VTC2__mannose-1-phosphate guanylyltransferase protein was commonly expressed under elevated CO_2_ and low O_2_–high CO_2_ treatments, indicating differential sensing, signalling, and stress responses in *P. patens* (Table 3). Together, microarray data indicate that moss gametophytes are likely to have experienced oxidative stress under elevated CO_2_ levels, which intensified transcriptional responses associated with the acquisition of abiotic stress tolerance.

**Table 3.** *Up-regulated* P. patens *genes involved in oxidative signalling and oxidative stress responses*

Gene ID	Annotation	*Arabidopsis* Gene Identifier	Sub-ambient O_2_	Elevated CO_2_	Low O_2_– high CO_2_
Pp1s34_208V6	L-galactono-1,4-lactone dehydrogenase	AT3G47930	3.12		-
Pp1s98_250V6	GDP-D-mannose 3\′,5\′-epimerase	AT5G28840	2.45	2.20	-
Pp1s396_10V6	SOUL haem-binding family protein	AT5G20140	2.80	2.70	-
Pp1s98_9V6	tetraticopeptide domain-containing thioredoxin	AT3G17880	-	2.54	-
Pp1s505_9V6	Rubredoxin-like superfamily protein	AT5G51010	-	2.61	-
Pp1s27_275V6	inositol monophosphatase family protein	AT3G02870	-	2.21	-
Pp1s404_1V6	microsomal glutathione s-transferase, putative	AT1G65820	-	2.54	-
Pp1s66_172V6	glutathione S-transferase PHI 9	AT2G30860	-	2.35	-
Pp1s182_83V6	microsomal glutathione s-transferase, putative	AT1G65820	-	2.21	-
Pp1s224_120V6	multidrug resistance-associated protein 2	AT2G34660	-	2.02	-
Pp1s21_97V6	12-oxophytodienoate reductase 2	AT1G76690	-	2.23	-
Pp1s326_44V6	12-oxophytodienoate reductase 1	AT1G76680	-	2.28	-
Pp1s184_82V6	peroxidase superfamily protein	AT5G06730	-	2.55	-
Pp1s114_207V6	myoinositol-1-phosphate guanylyltransferase	AT4G26850	-	2.25	4.22
Pp1s20_77V6	peroxidase superfamily protein	AT5G14130	-	-	2.17
Pp1s306_39V6	peroxidase superfamily protein	AT5G05340	-	-	2.91
Pp1s273_43V6	thioredoxin superfamily protein	AT1G07700	-	-	5.83
Pp1s106_67V6	thioredoxin superfamily protein	AT4G03520	-	-	2.42
Pp1s178_130V6	myoinositol-1-phosphate guanylyltransferase	AT4G26850	-	-	2.29
Pp1s71_207V6	hemoglobin 1	AT2G16060	-	-	2.51
Pp1s40_134V6	1-cysteine peroxiredoxin 1	AT1G48130	-	-	2.93
Pp1s98_113V6	copper chaperone for SOD1	AT1G12520	-	-	2.03
Pp1s223_74V6	catalase 1	AT1G20630	-	-	6.68
Pp1s506_15V6	GroES-like zinc-binding dehydrogenase family protein	AT5G43940	-	-	2.66

## Conclusion

Anthropogenic activities have contributed to accelerate the emission of CO_2_ into the atmosphere. Elevated CO_2_ is recognized as a major contributing factor to the effects that global climate change exerts on plants (IPCC, 2013). In the present study, changes in the transcriptome of gametophytes of the moss *P. patens* to differential CO_2_ to O_2_ concentrations have been profiled to understand moss acclimation responses to elevated CO_2_, sub-ambient O_2_, and low O_2_–high CO_2_ conditions at the molecular level. Microarray analyses showed that expression of *P. patens* genes related to CO_2_ fixation, RuBP regeneration, and starch synthesis was significantly altered, indicating photosynthetic acclimation of *P. patens* exposed to high CO_2_ to O_2_ concentrations. It is likely that elevated CO_2_ caused an accumulation of sugars and starch in *P. patens* as increased transcript abundance of genes encoding enzymes in starch synthesis were observed. Accumulation of soluble sugars may have also contributed to the decrease in RuBP and RuBP activase transcripts in *P. patens*. However, quantitative analysis of RuBP, chlorophyll, and sugars such as sucrose, fructose, and glucose under elevated CO_2_ must be conducted before definitive conclusions can be drawn. The result also indicated that elevated CO_2_ in the presence of ambient O_2_ and sub-ambient levels of O_2_ evoked large-scale transcriptional reprogramming of *P. patens* gametophytes, and changes in oxidative signalling and defence responses. Based on the transcriptome data, it is hypothesized that transcriptional reprogramming may reflect differences in the CO_2_ to O_2_ ratio of the imposed experimental atmospheres (Table 1). It will be interesting in future experiments to vary the concentrations of CO_2_ and O_2_ and examine the effects of a similar CO_2_ to O_2_ ratio for all three atmospheric conditions on the transcriptome. This will enable us to gain better insights into the transcriptional responses exhibited by *P. patens* gametophytes subjected to different atmospheric CO_2_ and O_2_ concentrations.

## Supplementary data

Fig. S1. MapMan molecular functional classification of *P. patens* genes (using *Arabidopsis* homologues as a reference) where the expression levels were significantly up- and down-regulated following a 7-day exposure to (A) sub-ambient O_2_ (up-regulated), (B) sub-ambient O_2_ (down-regulated), (C) elevated CO_2_ (up-regulated), (D) elevated CO_2_ (down-regulated), (E) Low O_2_–high CO_2_ (up-regulated), and (F) Low O_2_–high CO_2_ (down-regulated).

Fig. S2. Genes encoding transcriptional regulators that were (A) up- and (B) down-regulated in *P. patens* gametophytes subjected to by elevated CO_2_ (1500 ppmV), sub-ambient O_2_ (13%), and low O_2_–high CO_2_.

Fig. S3. Effects of elevated CO_2_, sub-ambient O_2_, and low O_2_–high CO_2_ treatment on expression of genes encoding proteins involved in CO_2_ fixation, RuBP regeneration, and starch synthesis in moss *P. patens* gametophytes. Venn diagram showing the number of commonly and distinctly expressed (up- and down-regulated) genes.

Fig. S4. Membrane transport-related genes that were (A) up- and (B) down-regulated in *P. patens* gametophytes subjected to by elevated CO_2_ (1500 ppmV), sub-ambient O_2_ (13%), and low O_2_–high CO_2_.

Table S1. List of probes and their respective primer sequences used for semi-quantitative RT-PCR.

Table S2. Significantly regulated *P. patens* genes encoding transcription factors following a 7-day exposure to sub-ambient O_2_ (13%), elevated CO_2_ (1500 ppmV), and low O_2_–high CO_2_ treatment.

Table S3. Effect of elevated CO_2_ or sub-ambient O_2_ individually or in combination (low O_2_–high CO_2_) on *P. patens* transcripts functionally associated with the photochemical reactions of photosynthesis.

Table S4A. Altered expression of highly expressed *P. patens* transcripts involved in signal transduction regulated by metabolic sugar in response to elevated CO_2_ or sub-ambient O_2_ individually or in combination (low O_2_–high CO_2_).

Table S4B. Regulation of P*. patens* transcripts involved in glycolysis under elevated CO_2_ or sub-ambient O_2_ individually or in combination (low O_2_–high CO_2_).

Table S5A. Highly induced *P. patens* transcripts functionally associated with membrane transport (based on MapMan analysis).

Table S5B. Significantly down-regulated *P. patens* transcripts functionally associated with membrane transport.

Table S6. Effect of differential CO_2_ to O_2_ levels on *P. patens* transcripts involved in hormone metabolism (based on MapMan analysis using *Arabidopsis* homologues).

Table S7. Effect of differential CO_2_ to O_2_ levels on expression of *P. patens* stress-associated transcripts (based on MapMan analysis using *Arabidopsis* homologues).

Supplementary Data
