# Supplementary material for: Genome-wide transcriptomic analysis of the effects of sub-ambient atmospheric oxygen and elevated atmospheric carbon dioxide levels on gametophytes of the moss, Physcomitrella patens
Source: J Exp Bot. 2015 May 6;66(13):4001–12. doi: 10.1093/jxb/erv197 (PMC4473992; doi:10.1093/jxb/erv197)
Supplement: Supplementary Data [file supp_66_13_4001__index.html]

Genome-wide transcriptomic analysis of the effects of sub-ambient atmospheric oxygen and elevated atmospheric carbon dioxide levels on gametophytes of the moss, Physcomitrella patens — Supplementary Data 

# Genome-wide transcriptomic analysis of the effects of sub-ambient atmospheric oxygen and elevated atmospheric carbon dioxide levels on gametophytes of the moss, *Physcomitrella patens*

## Supplementary Data

Data files

- Supplementary Data - Supplementary Data
- Supplementary Data - Supplementary Data
