# Supplementary material for: Genome-wide transcriptomic analysis of the effects of sub-ambient atmospheric oxygen and elevated atmospheric carbon dioxide levels on gametophytes of the moss, Physcomitrella patens
Source: J Exp Bot. 2015 May 6;66(13):4001–12. doi: 10.1093/jxb/erv197 (PMC4473992; doi:10.1093/jxb/erv197)

Supplementary Fig. S1. MapMan molecular functional classification of *P. patens* genes (using Arabidopsis homologues as a reference) where the expression levels were significantly up- and down-regulated following a 7-day exposure to (A) sub-ambient O_2 (up-regulated), (B) sub-ambient O_2 (down-regulated), (C) elevated CO_2 (up-regulated), (D) elevated CO_2 (down-regulated), (E) Low O_2- high CO_2 (up-regulated) and (F) Low O_2- high CO_2 (down-regulated). Molecular function class (on Y-axis) are organized in increasing order of the number genes (gene count on X-axis). Significance/ confidence (*p*-value) for each class is given in bracket. The values in the blue circle represents total number of genes with altered expression (with fold change (FC) ≥ 2), the values in red and green circles indicates the number of up or down regulated genes respectively. Values in empty circles with red and green border represent the number of corresponding *A. thaliana* locus IDs (AGIs) used for MapMan molecular functional classification (Provart and Zhu, 2003).

(A)

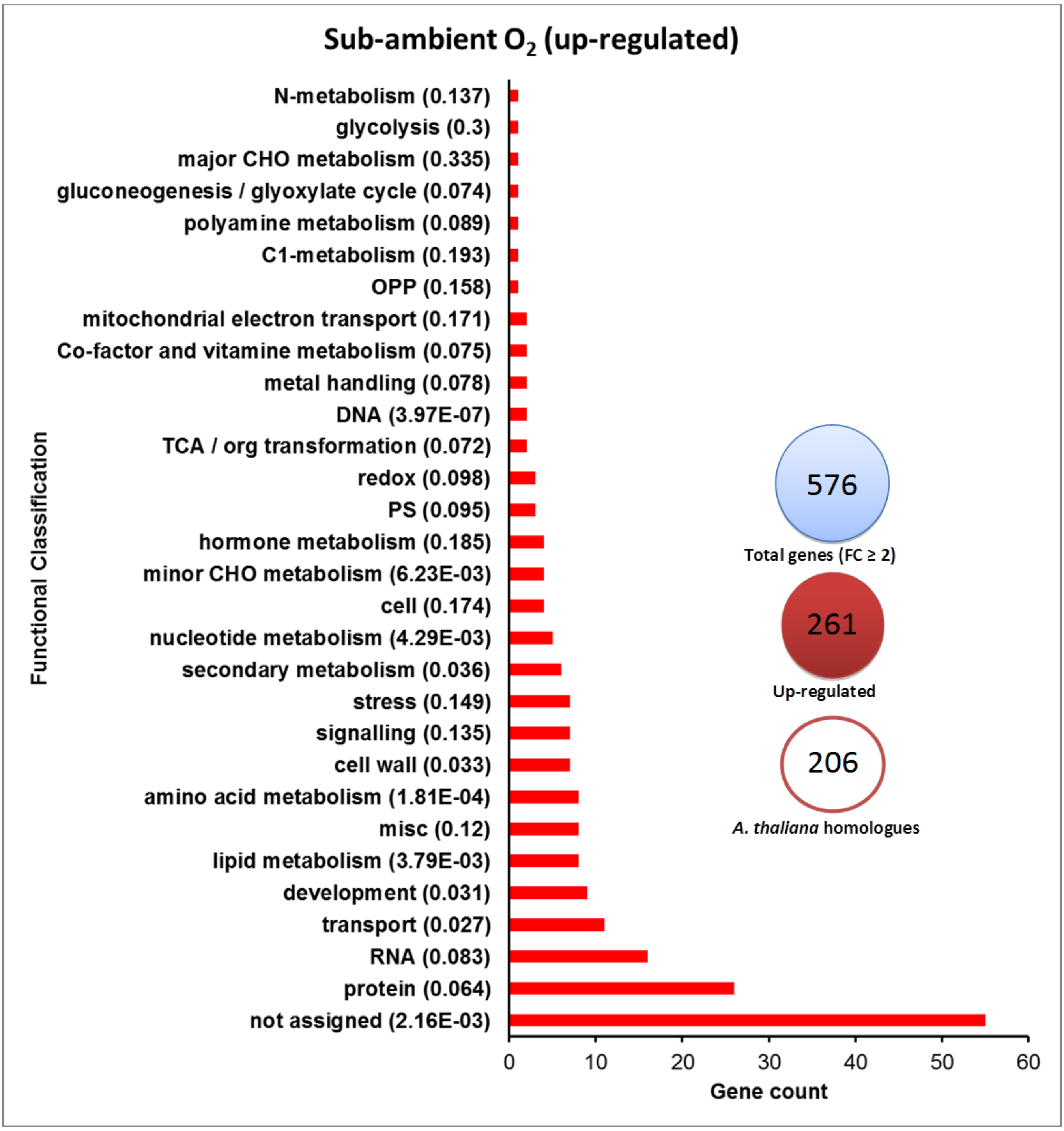
Sub-ambient O2 (up-regulated)
N-metabolism (0.137)
glycolysis (0.3)
major CHO metabolism (0.335)
gluconeogenesis / glyoxylate cycle (0.074)
polyamine metabolism (0.089)
C1-metabolism (0.193)
OPP (0.158)
mitochondrial electron transport (0.171)
Co-factor and vitamine metabolism (0.075)
metal handling (0.078)
DNA (3.97E-07)
TCA / org transformation (0.072)
redox (0.098)
PS (0.095)
hormone metabolism (0.185)
minor CHO metabolism (6.23E-03)
cell (0.174)
nucleotide metabolism (4.29E-03)
secondary metabolism (0.036)
stress (0.149)
signalling (0.135)
cell wall (0.033)
amino acid metabolism (1.81E-04)
misc (0.12)
lipid metabolism (3.79E-03)
development (0.031)
transport (0.027)
RNA (0.083)
protein (0.064)
not assigned (2.16E-03)
Functional Classification
0
10
20
30
40
50
60
Gene count
576
Total genes (FC ≥ 2)
261
Up-regulated
206
A. thaliana homologues

(B)

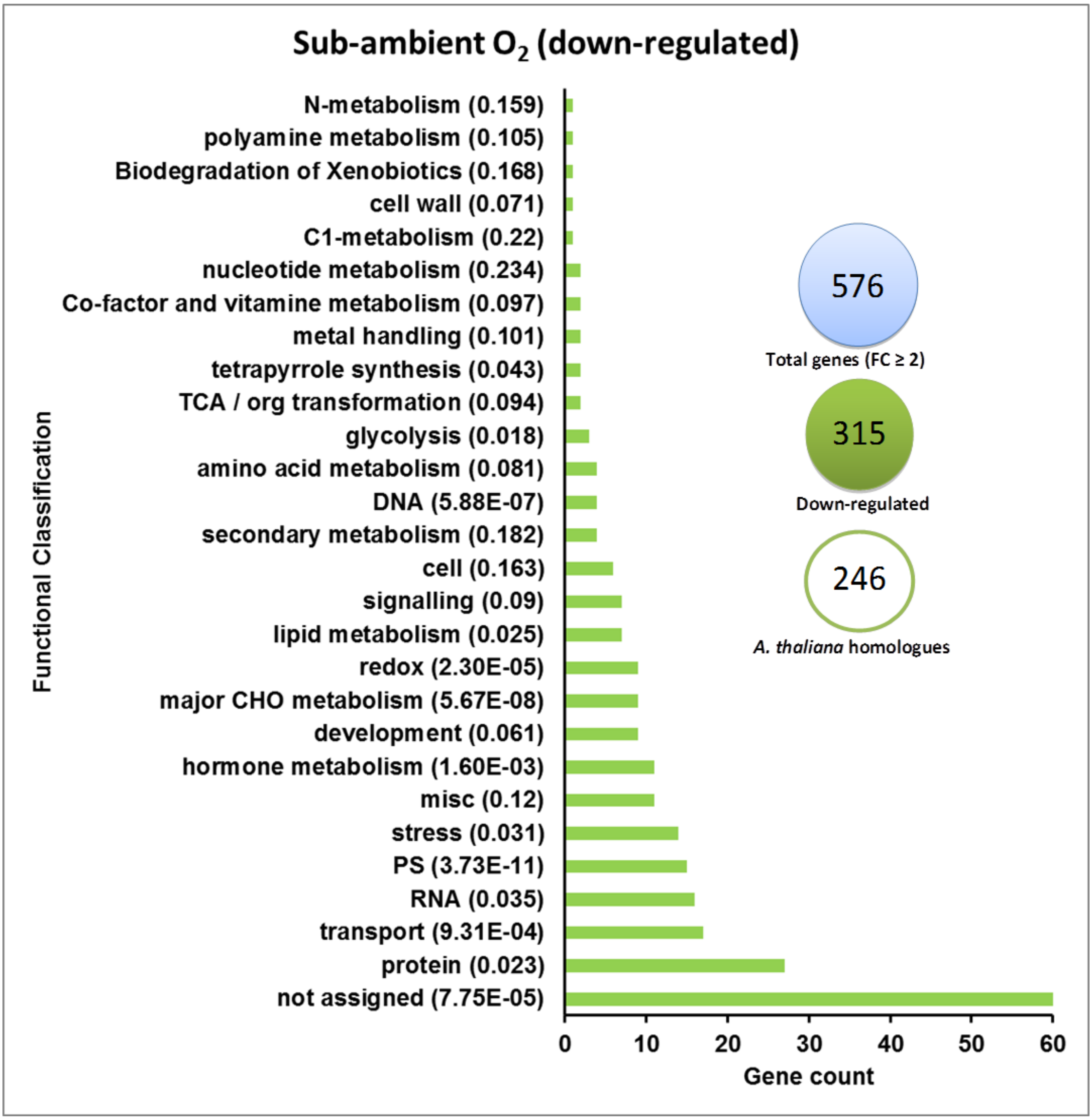

Sub-ambient O2 (down-regulated)
N-metabolism (0.159)
polyamine metabolism (0.105)
Biodegradation of Xenobiotics (0.168)
cell wall (0.071)
C1-metabolism (0.22)
nucleotide metabolism (0.234)
Co-factor and vitamine metabolism (0.097)
metal handling (0.101)
tetrapyrrole synthesis (0.043)
TCA / org transformation (0.094)
glycolysis (0.018)
amino acid metabolism (0.081)
DNA (5.88E-07)
secondary metabolism (0.182)
cell (0.163)
signalling (0.09)
lipid metabolism (0.025)
redox (2.30E-05)
major CHO metabolism (5.67E-08)
development (0.061)
hormone metabolism (1.60E-03)
misc (0.12)
stress (0.031)
PS (3.73E-11)
RNA (0.035)
transport (9.31E-04)
protein (0.023)
not assigned (7.75E-05)
Functional Classification
0
10
20
30
40
50
60
Gene count
576
Total genes (FC ≥ 2)
315
Down-regulated
246
A. thaliana homologues

(C)

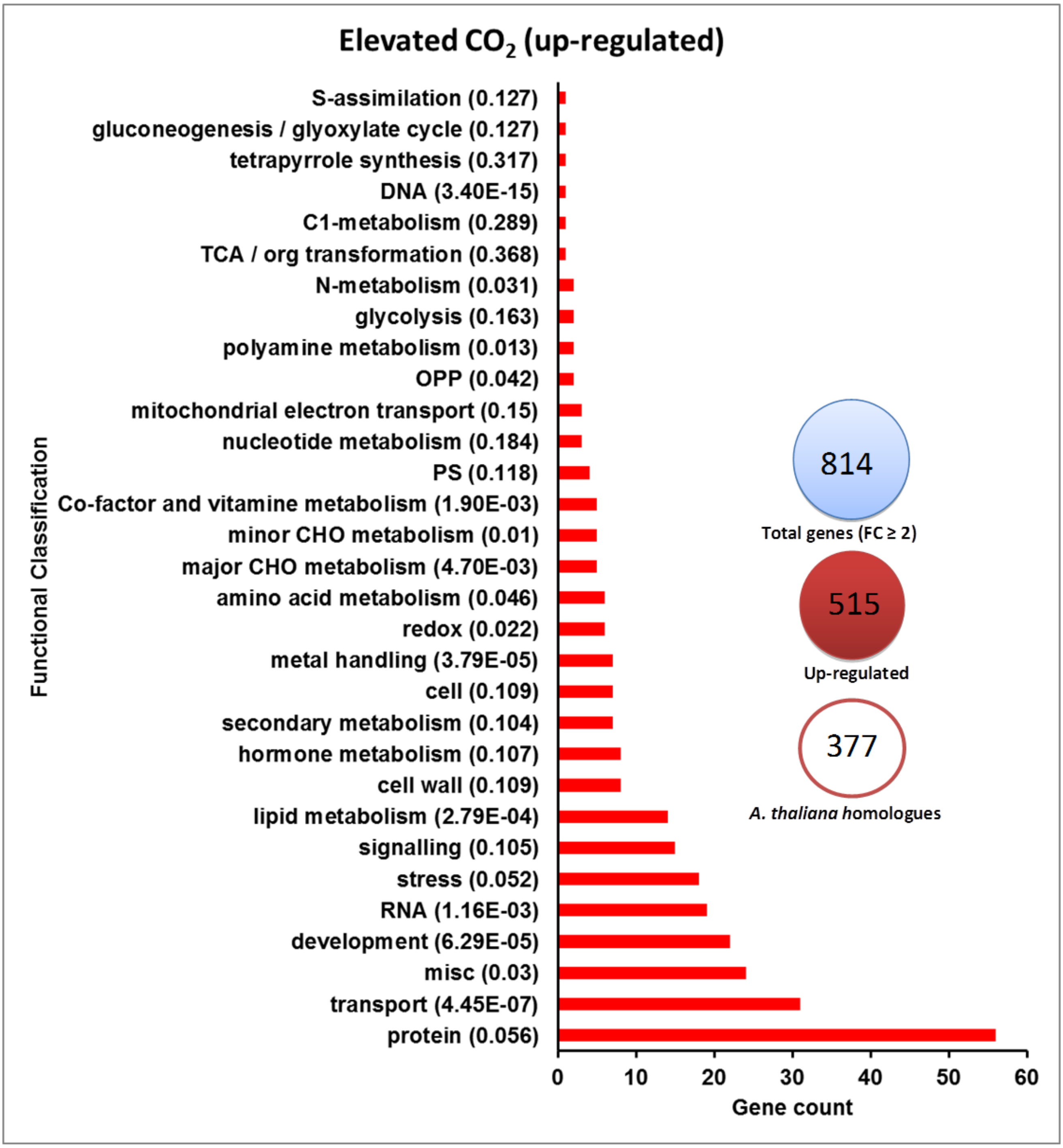

Elevated CO_2 (up-regulated)
Functional Classification
S-assimilation (0.127)
gluconeogenesis / glyoxylate cycle (0.127)
tetrapyrrole synthesis (0.317)
DNA (3.40E-15)
C1-metabolism (0.289)
TCA / org transformation (0.368)
N-metabolism (0.031)
glycolysis (0.163)
polyamine metabolism (0.013)
OPP (0.042)
mitochondrial electron transport (0.15)
nucleotide metabolism (0.184)
PS (0.118)
Co-factor and vitamine metabolism (1.90E-03)
minor CHO metabolism (0.01)
major CHO metabolism (4.70E-03)
amino acid metabolism (0.046)
redox (0.022)
metal handling (3.79E-05)
cell (0.109)
secondary metabolism (0.104)
hormone metabolism (0.107)
cell wall (0.109)
lipid metabolism (2.79E-04)
signalling (0.105)
stress (0.052)
RNA (1.16E-03)
development (6.29E-05)
misc (0.03)
transport (4.45E-07)
protein (0.056)
0
10
20
30
40
50
60
Gene count
814
Total genes (FC ≥ 2)
515
Up-regulated
377
A. thaliana homologues

(D)

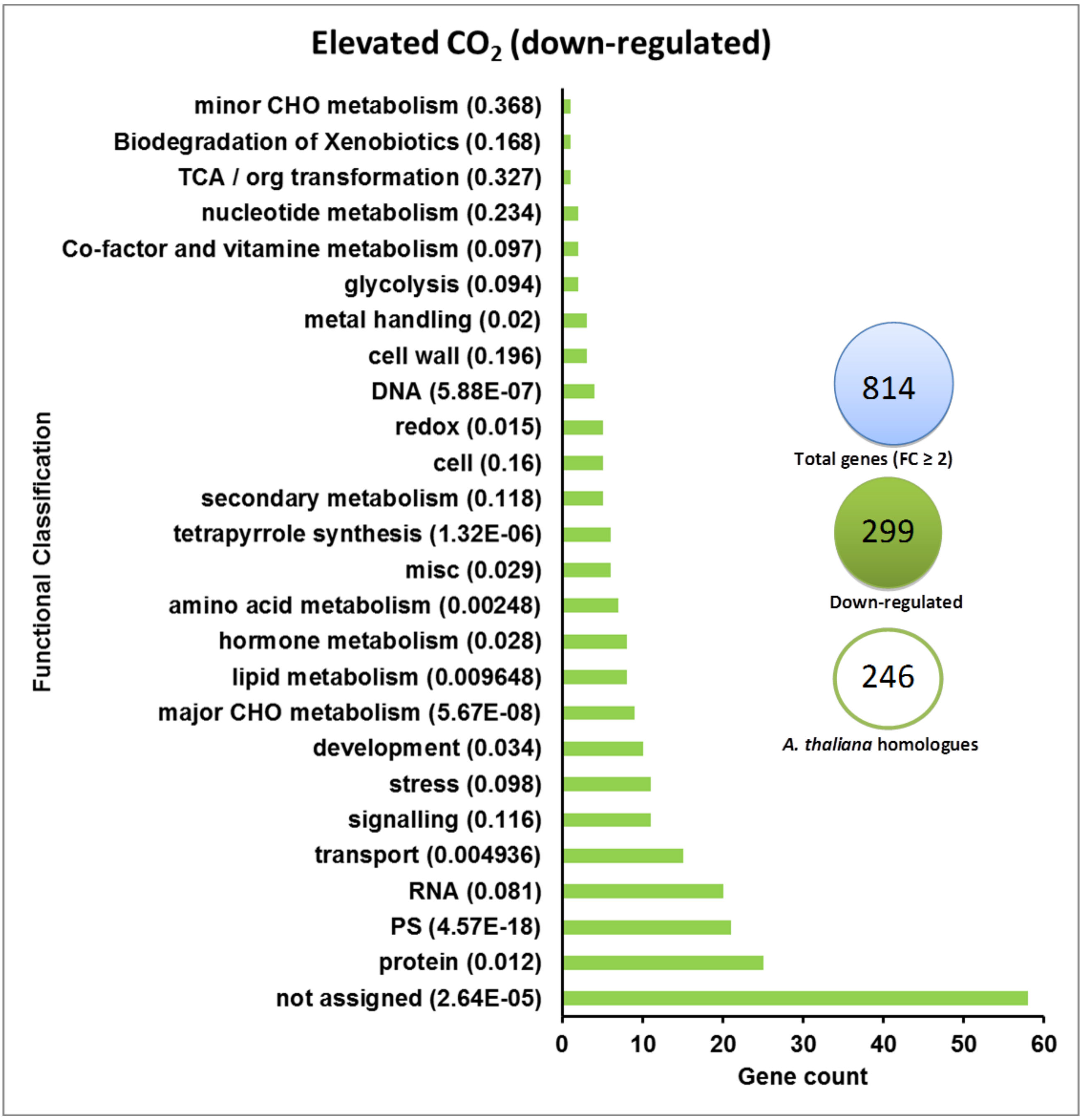

Elevated CO_2 (down-regulated)
minor CHO metabolism (0.368)
Biodegradation of Xenobiotics (0.168)
TCA / org transformation (0.327)
nucleotide metabolism (0.234)
Co-factor and vitamine metabolism (0.097)
glycolysis (0.094)
metal handling (0.02)
cell wall (0.196)
DNA (5.88E-07)
redox (0.015)
cell (0.16)
secondary metabolism (0.118)
tetrapyrrole synthesis (1.32E-06)
misc (0.029)
amino acid metabolism (0.00248)
hormone metabolism (0.028)
lipid metabolism (0.009648)
major CHO metabolism (5.67E-08)
development (0.034)
stress (0.098)
signalling (0.116)
transport (0.004936)
RNA (0.081)
PS (4.57E-18)
protein (0.012)
not assigned (2.64E-05)
Functional Classification
0
10
20
30
40
50
60
Gene count
814
Total genes (FC ≥ 2)
299
Down-regulated
246
A. thaliana homologues

(E)

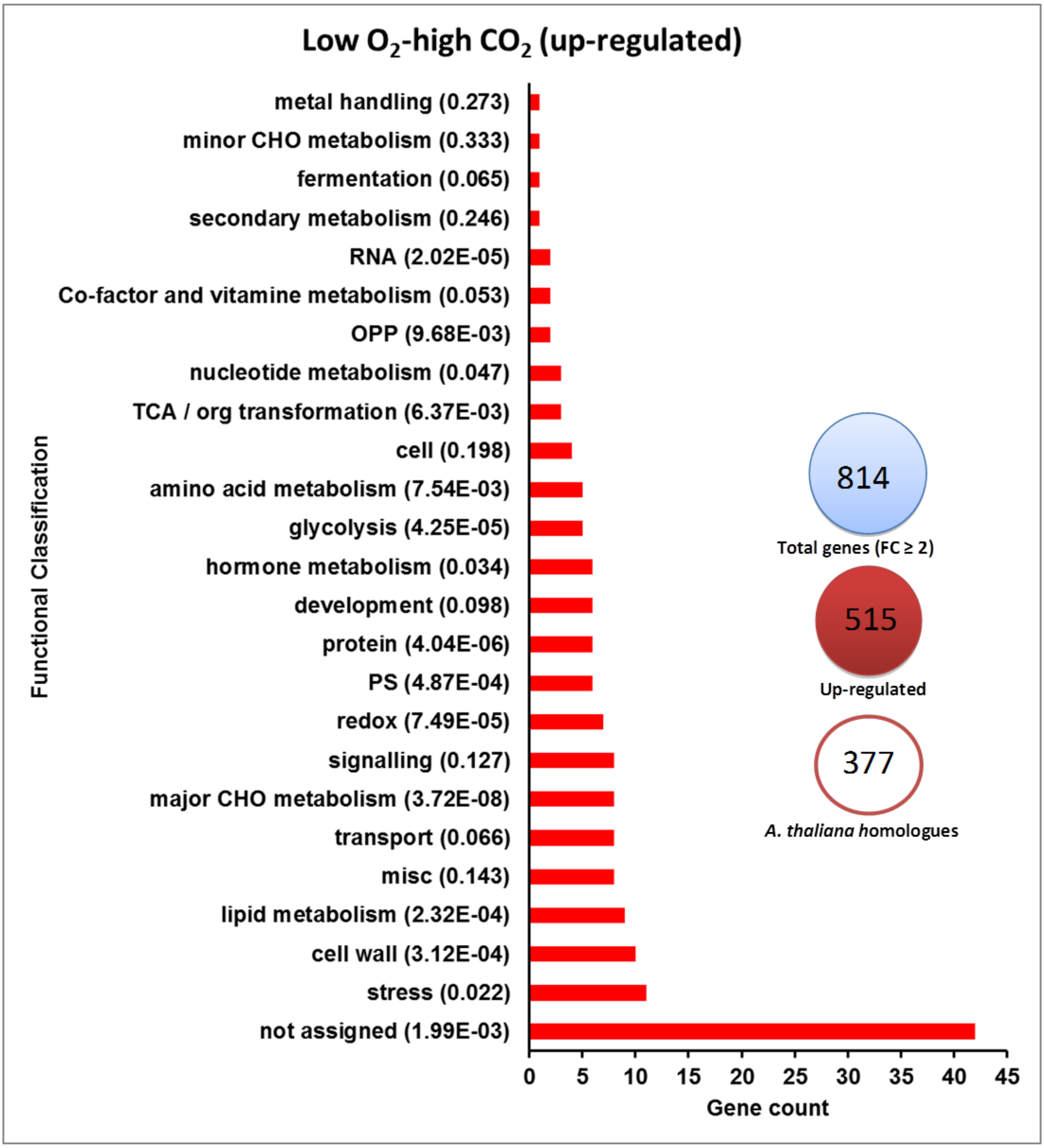

Low O_2-high CO_2 (up-regulated)
metal handling (0.273)
minor CHO metabolism (0.333)
fermentation (0.065)
secondary metabolism (0.246)
RNA (2.02E-05)
Co-factor and vitamine metabolism (0.053)
OPP (9.68E-03)
nucleotide metabolism (0.047)
TCA / org transformation (6.37E-03)
cell (0.198)
amino acid metabolism (7.54E-03)
glycolysis (4.25E-05)
hormone metabolism (0.034)
development (0.098)
protein (4.04E-06)
PS (4.87E-04)
redox (7.49E-05)
signalling (0.127)
major CHO metabolism (3.72E-08)
transport (0.066)
misc (0.143)
lipid metabolism (2.32E-04)
cell wall (3.12E-04)
stress (0.022)
not assigned (1.99E-03)
Functional Classification
0
5
10
15
20
25
30
35
40
45
Gene count
814
Total genes (FC ≥ 2)
515
Up-regulated
377
A. thaliana homologues

(F)

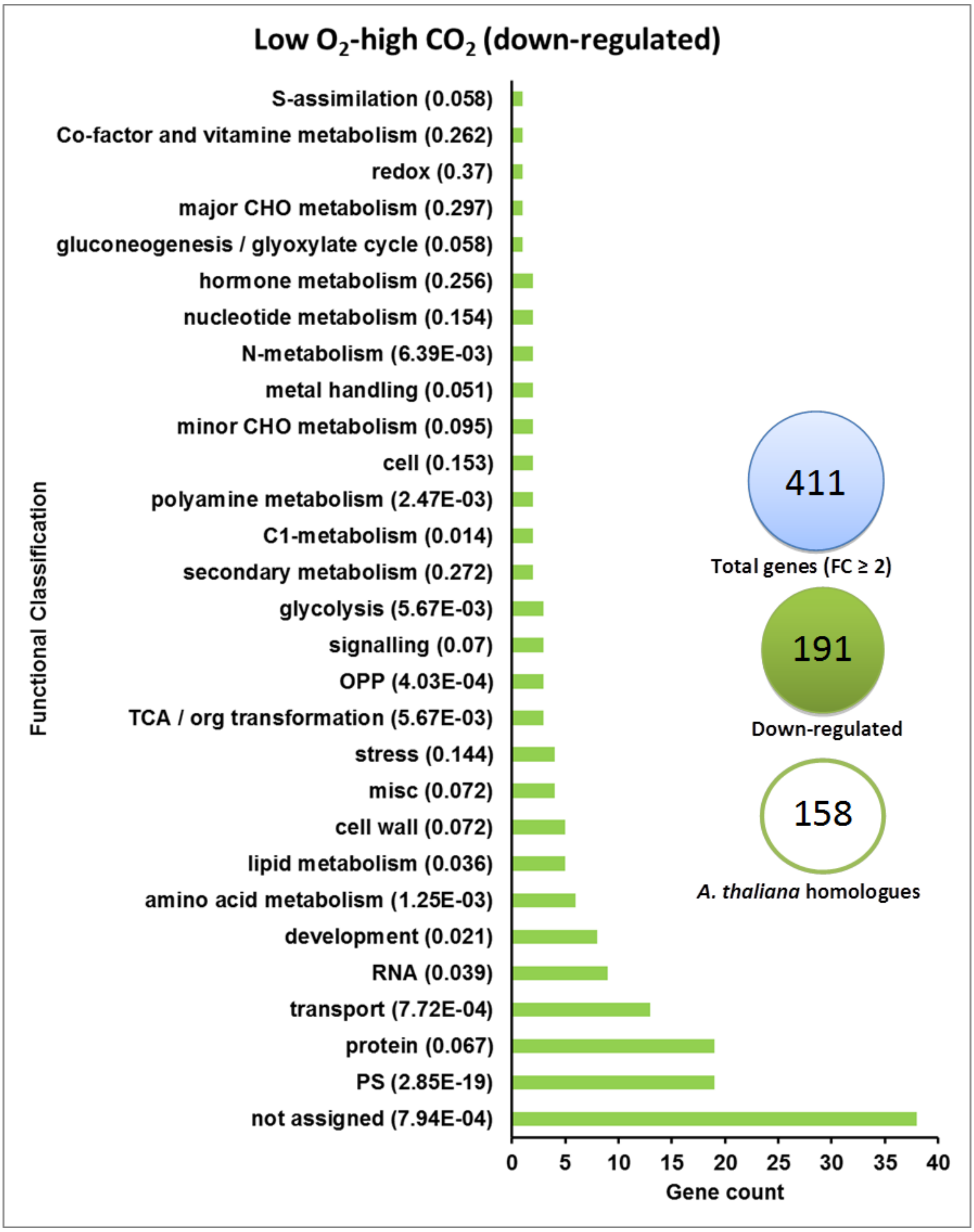
Low O_2-high CO_2 (down-regulated)
S-assimilation (0.058)
Co-factor and vitamine metabolism (0.262)
redox (0.37)
major CHO metabolism (0.297)
gluconeogenesis / glyoxylate cycle (0.058)
hormone metabolism (0.256)
nucleotide metabolism (0.154)
N-metabolism (6.39E-03)
metal handling (0.051)
minor CHO metabolism (0.095)
cell (0.153)
polyamine metabolism (2.47E-03)
C1-metabolism (0.014)
secondary metabolism (0.272)
glycolysis (5.67E-03)
signalling (0.07)
OPP (4.03E-04)
TCA / org transformation (5.67E-03)
stress (0.144)
misc (0.072)
cell wall (0.072)
lipid metabolism (0.036)
amino acid metabolism (1.25E-03)
development (0.021)
RNA (0.039)
transport (7.72E-04)
protein (0.067)
PS (2.85E-19)
not assigned (7.94E-04)
Functional Classification
0
5
10
15
20
25
30
35
40
Gene count
411
Total genes (FC ≥ 2)
191
Down-regulated
158
A. thaliana homologues

Supplementary Fig. S2. Genes encoding transcriptional regulators that were (A) up- and (B) down-regulated in *P. patens* gametophytes subjected to by elevated CO_2 (1500 ppmV), sub-ambient O_2 (13%), and low O_2- high CO_2.

(A) Number of up-regulated *P. patens* genes

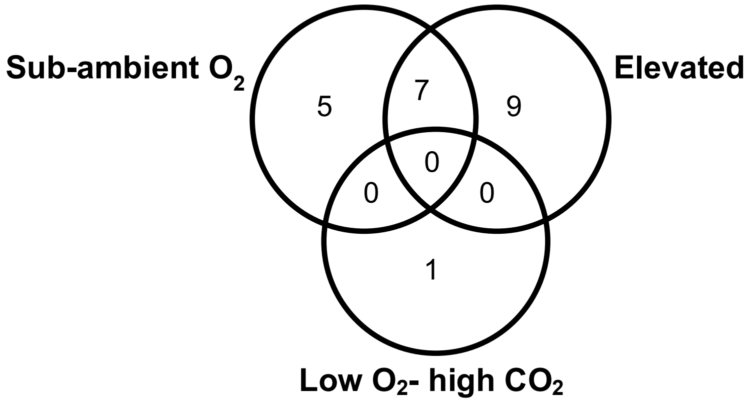

(B) Number of down-regulated *P. patens* genes

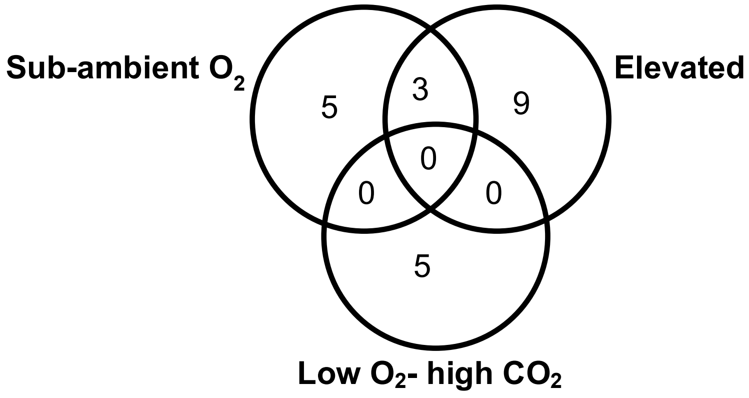

Supplementary Fig. S3. Effects of elevated CO_2, sub-ambient O_2 and low O_2-high CO_2 treatment on expression of genes encoding proteins involved in CO_2 fixation, RuBP regeneration and starch synthesis in moss *P. patens* gametophytes. Venn diagram showing the number of commonly and distinctly expressed (up and down-regulated) genes.

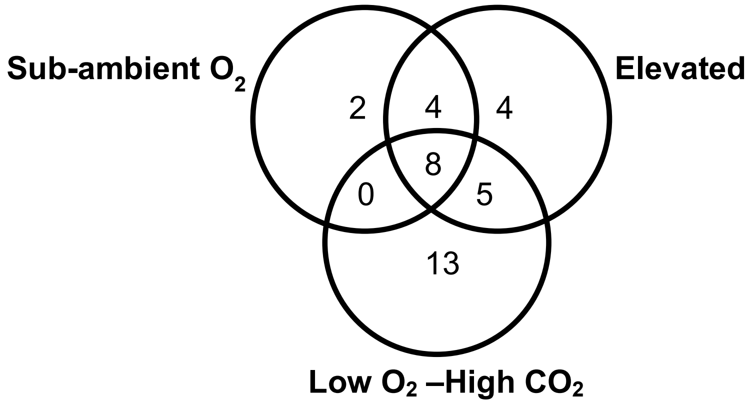

Supplementary Fig. S4. Membrane transport-related genes that were (A) up- and (B) down-regulated in *P. patens* gametophytes subjected to by elevated CO_2 (1500 ppmV), sub-ambient O_2 (13%), and low O_2- high CO_2.

(A) Number of up-regulated *P. patens* genes

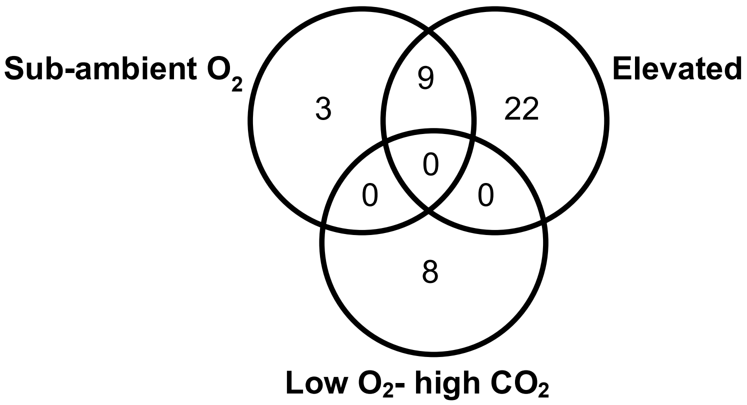

(B) Number of down-regulated *P. patens* genes

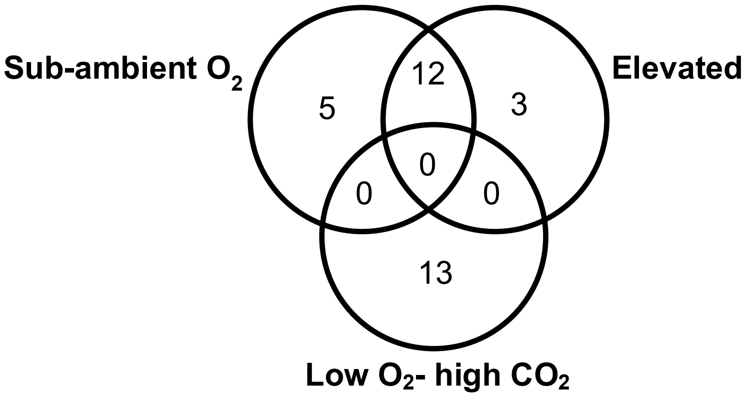

Supplement: Supplementary Data [file supp_erv197_erv197_supplementray_files.pdf]
